# Metagenomic and chemical characterization of soil cobalamin production

**DOI:** 10.1038/s41396-019-0502-0

**Published:** 2019-09-06

**Authors:** Xinda Lu, Katherine R. Heal, Anitra E. Ingalls, Andrew C. Doxey, Josh D. Neufeld

**Affiliations:** 1 Department of Biology, University of Waterloo, Waterloo, ON Canada; 2 School of Oceanography, University of Washington, Seattle, WA USA; 3 Present Address: Department of Civil and Environmental Engineering, Massachusetts Institute of Technology, Cambridge, MA USA

**Keywords:** Metagenomics, Soil microbiology

## Abstract

Cobalamin (vitamin B_12_) is an essential enzyme cofactor for most branches of life. Despite the potential importance of this cofactor for soil microbial communities, the producers and consumers of cobalamin in terrestrial environments are still unknown. Here we provide the first metagenome-based assessment of soil cobalamin-producing bacteria and archaea, quantifying and classifying genes encoding proteins for cobalamin biosynthesis, transport, remodeling, and dependency in 155 soil metagenomes with profile hidden Markov models. We also measured several forms of cobalamin (CN-, Me-, OH-, Ado-B_12_) and the cobalamin lower ligand (5,6-dimethylbenzimidazole; DMB) in 40 diverse soil samples. Metagenomic analysis revealed that less than 10% of soil bacteria and archaea encode the genetic potential for de novo synthesis of this important enzyme cofactor. Predominant soil cobalamin producers were associated with the *Proteobacteria*, *Actinobacteria*, *Firmicutes*, *Nitrospirae*, and *Thaumarchaeota*. In contrast, a much larger proportion of abundant soil genera lacked cobalamin synthesis genes and instead were associated with gene sequences encoding cobalamin transport and cobalamin-dependent enzymes. The enrichment of DMB and corresponding DMB synthesis genes, relative to corrin ring synthesis genes, suggests an important role for cobalamin remodelers in terrestrial habitats. Together, our results indicate that microbial cobalamin production and repair serve as keystone functions that are significantly correlated with microbial community size, diversity, and biogeochemistry of terrestrial ecosystems.

## Introduction

**Fig. 1:**
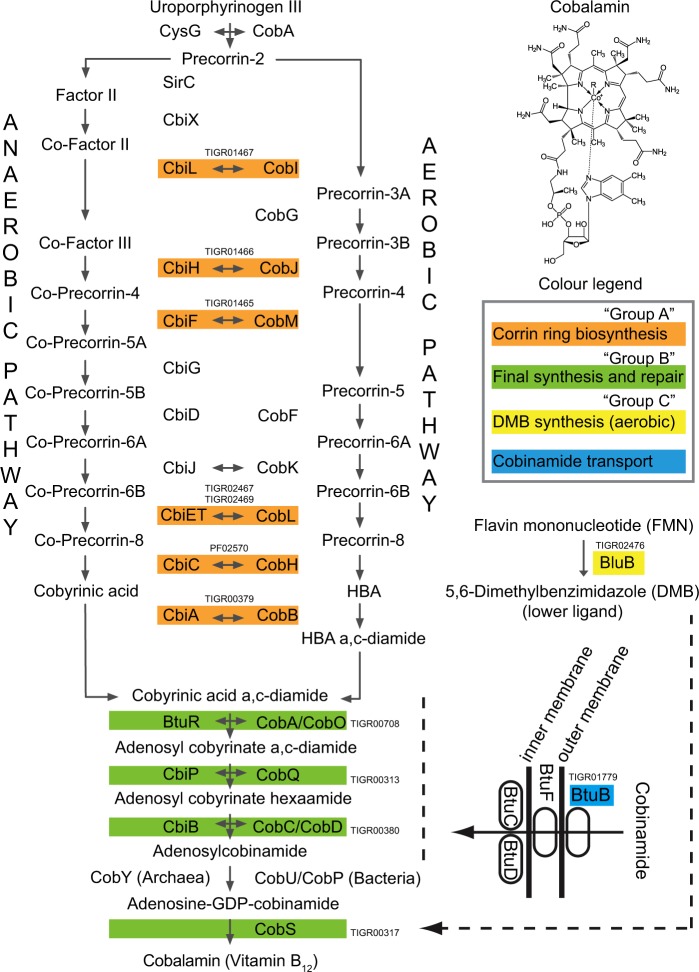
Cobalamin biosynthesis and transport pathways, separating anaerobic and aerobic pathways with early and late incorporation of cobalt, respectively. Adapted from Doxey et al. [15] and Fang et al. [86]. Sequence homology and functional equivalency between aerobic and anaerobic pathway enzymes are indicated by horizontal arrows. Colors denote the groups of profile HMMs (see TIGR or PFAM HMM numbers adjacent to corresponding pathway steps) that contribute to various stages of cobalamin synthesis and/or salvage transport

Cobalamin (vitamin B_12_), once referred to as “nature’s most beautiful cofactor” [1], plays an important role as a coenzyme involved in the synthesis of nucleotides and amino acids, in addition to carbon processing and gene regulation within all domains of life [2, 3]. Despite widespread metabolic dependency on cobalamin, only a relatively small subset of bacteria and archaea are capable of its production [4–6]. Cobalamin is present across natural systems in several chemical forms that differ in their upper ligand, including the enzymatically active forms of adenosylcobalamin (Ado-B_12_), methylcobalamin (Me-B_12_), hydroxocobalamin (OH-B_12_), and the inactivated form cyanocobalamin (CN-B_12_), of which the upper ligands are interchangeable through both enzymatic and abiotic processes [4, 7]. Cobalamin biosynthesis requires more than 30 enzymatic steps, via aerobic or anaerobic pathways [8–10] (Fig. 1), and represents a high genomic and metabolic burden for microbial producers.

Previous research on cobalamin production and its environmental significance has focused on marine systems where many eukaryotic primary producers are limited by the availability of this short-lived cofactor [11], demonstrating a significant role for cobalamin in controlling marine microbial community composition and productivity [11–22]. Metagenomic, whole genome, and biochemical analyses revealed that taxa affiliated with *Proteobacteria* and *Thaumarchaeota* are major marine cobalamin producers [15, 19], whereas marine *Cyanobacteria* produce pseudocobalamin, a closely related compound with a lower ligand substituted by adenine [19]. Recent understanding of marine cobalamin and pseudocobalamin was furthered by methodological advances enabling the direct measurement of cobalamin in environmental samples [23]. Together, the availability of metagenomic data and advances in analytical chemistry techniques provide an ideal framework for exploring microbial cobalamin production, consumption, exchange, and interdependencies in terrestrial ecosystems.

Soils harbor high densities of microbial biomass and are among the most diverse microbial community ecosystems on Earth [24–26]. The majority of soil biogeochemical processes are mediated by microorganisms [27] and the sustainability of agricultural soils relies on microbial communities that help mediate nutrient supplies to crops [28]. Therefore, elucidating factors that influence soil microbial diversity, activity, and physiology help understand controls on terrestrial biogeochemical functions [29, 30]. As a cofactor required by a majority of microorganisms [31], cobalamin availability and distribution in soils are constrained by microbial producers, which may have profound and unexplored impacts on terrestrial biogeochemical cycles. Because cobalamin-dependent enzymes include ribonucleotide reductase [32], methyltransferases [33], and reductive dehalogenases [34], cobalamin availability governs a wide range of microbial processes, such as DNA replication and repair [35, 36], regulation of gene expression [37], amino acid synthesis [38], CO_2_ fixation [39], recycling of carbon to the tricarboxylic acid (TCA) cycle [13], and aromatic compound detoxification [40].

Nearly seventy years ago, microbial cultivation efforts showed that a high proportion of cultured soil bacteria rely on exogenous cobalamin [41–43], with the implication that a cohort of soil microorganisms must serve as in situ sources of this essential cofactor. Since these early studies, there has been a near-complete lack of research into the microbiology of soil cobalamin production, presumably due to methodological limitations. To address this knowledge gap, we identify and quantify representative marker genes encoding enzymes that are broadly distributed throughout the cobalamin-producing pathway in soil metagenomes with profile hidden Markov models (HMMs) and relate the distribution and taxonomy of genes encoding cobalamin biosynthesis proteins to genes encoding cobalamin transport and salvage proteins. We adapt a selection of representative marker genes (*cob*/*cbi*/*bluB*) for the cobalamin biosynthesis pathway from a previous study of cobalamin production in the marine environment (Fig. 1; Supplementary information S1) [14]. In addition, we survey for the cobalamin transporter gene, *btuB*, encoding a TonB-dependent outer membrane cobalamin receptor and transporter [44, 45], with a basic architecture similar to that of iron siderophore transporters [46]. In order to examine the use of cobalamin, we also analyzed the distribution of several genes encoding cobalamin-dependent enzymes [2]. Because many microorganisms require cobalamin as a cofactor, we tested the hypothesis that there would be a correlation between the relative abundances of cobalamin consumers and producers/remodelers. In addition to metagenomic analyses, we quantified and characterized the standing stock of in situ cobalamin in representative soil samples and assessed potential links between cobalamin concentration and soil microbial community abundance.

## Results

### Soil metagenomic survey of cobalamin producers

**Fig. 2:**
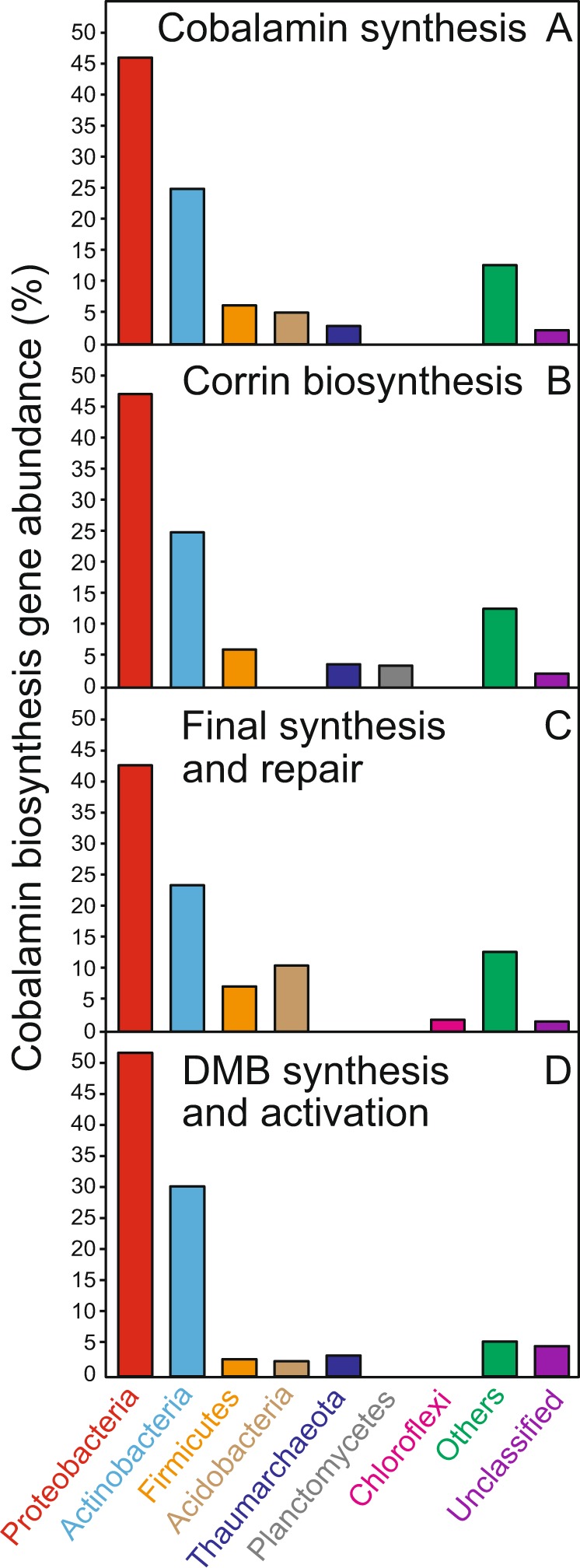
Taxonomic composition among all 155 soil metagenomes for **a** all 12 cobalamin biosynthesis genes, **b** corrin ring biosynthesis genes, **c** final synthesis and repair genes, and **d** the DMB synthesis and activation genes. Only the top five phyla are shown individually, other phyla are collectively denoted as “others”

Using selected profile HMMs (Fig. 1; Supplementary material S1) and translated nucleic acid sequences, we analyzed 155 soil metagenomes from diverse geographical locations and land use types (Supplementary material S1) to assess the contribution of different taxa to cobalamin-production potential via cobalamin biosynthesis (*cob*/*cbi*/*bluB*) gene taxonomic profiles (Fig. 2). Sequences affiliated with the *Proteobacteria* phylum dominated the genetic potential to produce cobalamin, contributing 45.9% of cobalamin biosynthesis genes across all soil metagenomes, followed by *Actinobacteria* (24.9%), *Firmicutes* (6.2%), *Acidobacteria* (5.1%), and *Thaumarchaeota* (2.9%) (Fig. 2a). These phyla contributed similarly to each of the cobalamin biosynthesis gene groups that are defined based on the cobalamin biosynthesis pathway (Fig. 1; Supplementary material S1): corrin ring biosynthesis protein coding genes (“Group A”; seven genes), final synthesis and repair protein coding genes (“Group B”; four genes), and 5,6-dimethylbenzimidazole (DMB) synthase coding gene (“Group C”; one gene; *bluB*) (Fig. 2b–d) [47, 48]. Soil type influenced the composition of potential cobalamin-producing taxa (permutational multivariate analysis of variance, *p* < 0.01), and cobalamin biosynthesis gene relative abundance from each major potential cobalamin-producing phylum (*Proteobacteria*, *Actinobacteria*, *Firmicutes*, *Acidobacteria*, and *Thaumarchaeota*) varied significantly among different soil types (ANOVA; *p* < 0.01; Fig. S1). On average, proteobacterial cobalamin biosynthesis genes dominated, accounting for ~55% of cobalamin synthesis genes detected in forest, herb, and wetland soil metagenomes, whereas proteobacterial proportions were lowest in desert soils (~20%; Fig. S1).

Several genera were associated with cobalamin synthesis based on the average relative abundance of HMM hits. For example, *Streptomyces* contributed the most to overall group A cobalamin biosynthesis genes (4.2%), followed by *Bradyrhizobium* (3.2%) and *Nitrososphaera* (2.8%) (Fig. S2A). Classifications of *Bradyrhizobium* (3.1%), *Acidobacterium* (2.9%), and *Candidatus* Koribacter (2.5%) were detected as the top three genera contributing to final synthesis and repair (Fig. S2B). The synthesis of DMB was attributed primarily to *Bradyrhizobium* (6.1%), *Mycobacterium* (4.8%), *Streptomyces* (4.5%), and *Nitrososphaera* (2.1%) (Fig. S2C). When evaluating the corresponding *rpoB* gene abundances for these same genera, 23.3% of the total soil microbial community, based on HMM hits to metagenomic sequence data, encoded corrin ring biosynthesis, 26.3% encoded final synthesis and repair, and 56.7% were associated with DMB production (Table 1).

**Table 1:** Relative abundances (evaluated by *rpoB* gene) of potential cobalamin-producing genera (*cob*/*cbi*/*bluB* gene associated) and potential cobalamin-dependent genera (*btuB* genes associated) in each of the three abundance categories (rare biosphere, intermediate abundance, and dominant taxa). Group A is corrin ring biosynthesis, Group B is final synthesis and repair, and Group C is 5,6-dimethylbenzimidazole (DMB) synthesis (Fig. 1). Only annotated genera are included in this table

Abundance category	Relative abundance of potential cobalamin- producing genera (%)			Relative abundance of potential cobalamin-dependent genera (%)
	Group A	Group B	Group C	
Rare	1.1	1.1	8.2	9.9
Intermediate	15.3	18.6	40.2	44.3
Dominant	6.9	6.6	8.3	13.2
Total	23.3	26.3	56.7	67.4

**Fig. 3:**
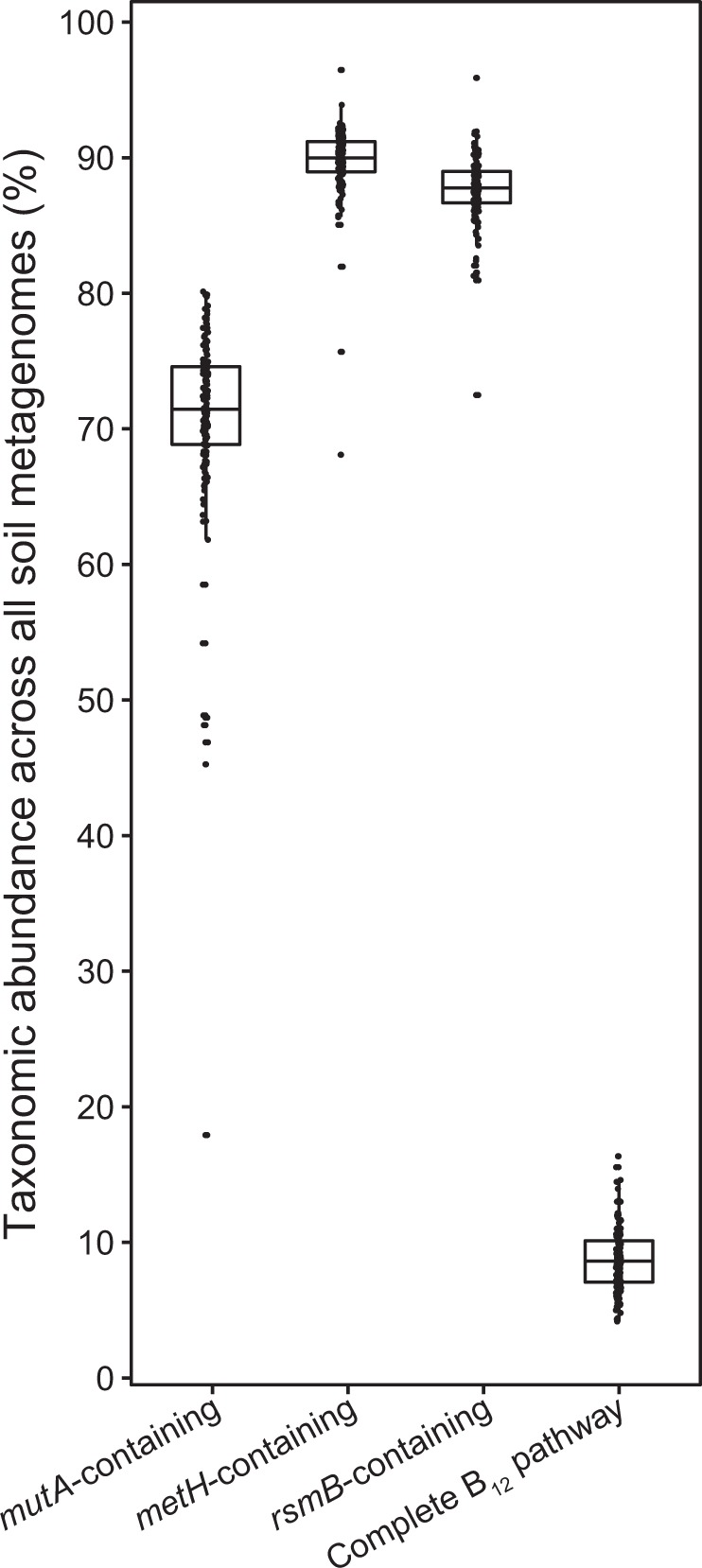
Proportion of genera encoding three cobalamin-dependent genes (i.e., *mutA*, *metH*, and *rsmB*) and those encoding the complete cobalamin biosynthesis pathway. Relative abundances were calculated as ratios to total *rpoB* gene abundances across all 155 soil metagenomes

Among all sampled metagenomes, 26 genera from 5 phyla (i.e., *Proteobacteria*, *Actinobacteria*, *Firmicutes*, *Nitrospirae*, and *Thaumarchaeota*) were associated with all 12 representative cobalamin biosynthesis genes (hereafter referred to as “complete”; Fig. S3). Together, these microorganisms with the complete genomic potential to produce cobalamin constituted 8.7 ± 2.3% of the global soil microbial community based on metagenome datasets, estimated by comparison to *rpoB* gene relative abundances of these same genera (Fig. 3 and S3). Of these, only three genera exceeded 1% average abundance (*Solirubrobacter*, 2.0%; *Bradyrhizobium*, 1.2%; *Streptomyces*, 1.1%). The number of genera with the potential for complete cobalamin synthesis showed a relationship with soil type (one-way ANOVA, *p* < 0.001, Fig. S4).

### Soil cobalamin producers and transporters

**Fig. 4:**
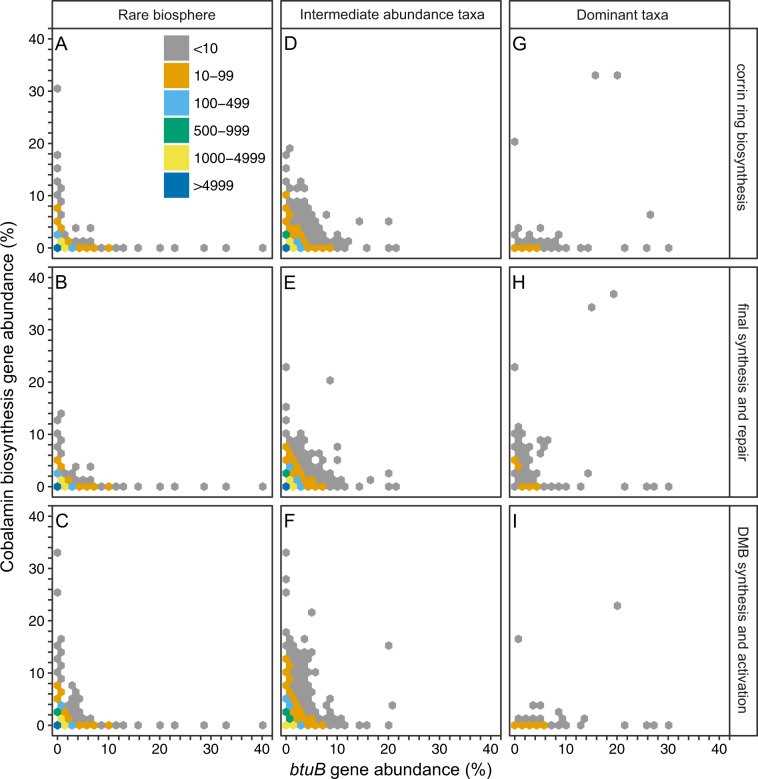
Relationship between genes coding for cobalamin transport (*btuB*) and synthesis enzymes (*cob*/*cbi*/*bluB*) across 155 soil metagenomes. Color denotes the number of genera occurring with a given *cob*/*cbi*/*bluB* and *btuB* gene relative abundance. **a**–**c** represent a genus from the rare biosphere (*rpoB* gene relative abundances were below 0.1%), **d**–**f** intermediate abundance taxa (*rpoB* gene relative abundance between 0.1 and 5%), and **g**–**i** dominant taxa (*rpoB* gene relative abundance over 5%). Mutual exclusion between the cobalamin transporter encoding gene (*btuB*) and cobalamin synthesis enzyme genes (*cob*/*cbi*/*bluB*) were tested with 10^5^ permutations, and significant (*p* < 0.05) for each gene group and either rare biosphere or intermediate abundance taxa. From top to bottom: Group A, corrin ring biosynthesis; Group B, final synthesis and repair; Group C, 5,6-dimethylbenzimidazole (DMB) synthesis

Across soil metagenomes, cobalamin-production and transport potential was evaluated by the relative abundance of *cob*/*cbi*/*bluB* and *btuB* genes, respectively. When examining the relationship between the relative abundance of cobalamin-producing enzyme coding genes (*cob*/*cbi* and *bluB*) and the cobalamin transporter protein coding gene (*btuB*) at the genus level across soil metagenomes, we observed mutual exclusion (permutation test *p* < 0.001) of these two gene complements for both rare biosphere members (Fig. 4a–c) and intermediate abundance taxa (between 0.1 and 5%; Fig. 4d–f). Thus, genera that were more represented among the cobalamin-synthesis enzyme coding gene pool were less well represented among the cobalamin transporter gene pool and vice versa. In general, dominant taxa encoded cobalamin transport potential while lacking genes associated with cobalamin production (Fig. 4g–i). However, there were several dominant (<10) genera that were affiliated with both cobalamin production and transport genes (Fig. 4g–i). The rare biosphere, as a whole, contributed to over half of the *btuB* gene abundance across all metagenomes, although these rare taxa collectively showed more cobalamin-producing potential than transport potential individually (Table 2).

**Table 2:** Relative contributions of the three microbial abundance categories (rare biosphere, intermediate abundance, and dominant genera) to cobalamin synthesis or transport (evaluated by *cob*/*cbi*/*bluB* or *btuB* gene, respectively). Relative contributions are evaluated by the proportion of genes (*cob*/*cbi*/*bluB* or *btuB*) in the metagenomes within each category. The rare biosphere refers to genera whose individual relative abundances are less than 0.1%; intermediate abundance genera: 0.1–5%; dominant taxa: >5%. Group A is corrin ring biosynthesis, Group B is final synthesis and repair, and Group C is 5,6-dimethylbenzimidazole (DMB) synthesis (Fig. 1). Only annotated genera are included in the three categories (rare, intermediate, and dominant) in this table. Proportions for unclassified genera are shown at the bottom

Abundance category	Group A (%)	Group B (%)	Group C (%)	*btuB* (%)
Rare	45.4	45.4	41.9	50.5
Intermediate	50.9	49.0	51.7	34.7
Dominant	1.0	3.6	0.9	3.6
Unclassified	2.7	2.0	5.5	11.2

Genes encoding cobalamin-dependent enzymes (methionine synthase, *metH*; methylmalonyl-CoA mutase, *mutA*; ribosomal small subunit methyltransferase, *rsmB*) were also surveyed among all 155 soil metagenomes to evaluate potential demand for cobalamin by genera that either produce (with enzymes encoded by *cob*/*cbi* and *bluB* genes) or transport (with transporter encoded by *btuB* gene) this cofactor (Supplementary material S1). Based on global soil metagenomes, genera affiliated with selected cobalamin-dependent enzyme genes (i.e., *metH*, *rsmB*, and *mutA*) accounted for over 70% of the total *rpoB*-encoding community (Fig. 3). The total encoded potential for cobalamin use, measured as the total abundance of genes encoding cobalamin-dependent enzymes, was significantly greater (paired *t*-test, *p* < 0.001) than the encoded potential for cobalamin production and transport (i.e., sum of *cob*/*cbi*/*bluB* and *btuB* gene abundance; Fig. S5).

### Soil cobalamin measurements

**Fig. 5:**
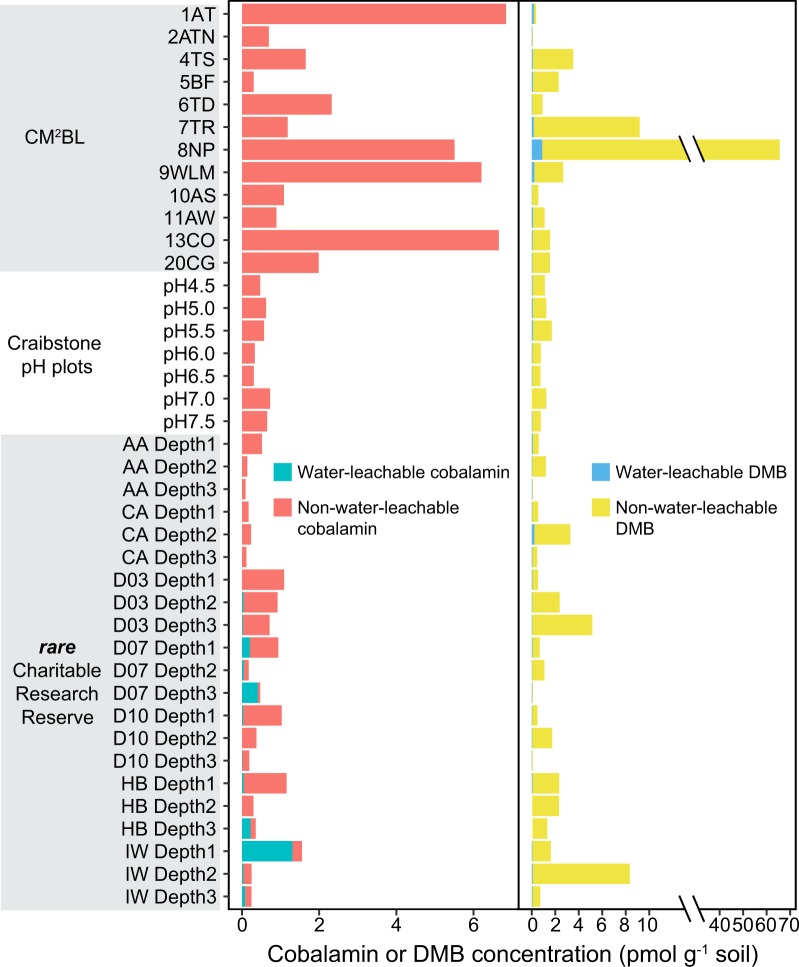
Concentration of total cobalamin (sum of both water-leachable and non-water-leachable cobalamin) and 5,6-dimethylbenzimidazole (DMB) measured in soils collected from the Canadian MetaMicroBiome Library (CM^2^BL) [82], the ***rare*** Charitable Research Reserve [83], and the Craibstone pH plots [84]. Water-leachable cobalamin and DMB only accounted for a small portion of total cobalamin and DMB. 1AT: Arctic Tundra; 2ATN: Arctic Tundra; 4TS: Tar sand; 5BF: Boreal coniferous forest; 6TD: Temperate deciduous forest; 7TR: temperate rain forest; 8NP: Northern peatlands; 9WLM: Wetland soil; 10AS: Agricultural soil (soy); 11AW: Agricultural soil (wheat); 13CO: Compost. AA: rare active agricultural site; D03: rare decommissioned agricultural (since 2003) site; D07: rare decommissioned agricultural (since 2007) site; D10: rare decommissioned agricultural (since 2010) site; CA: rare forest site; HB: rare forest site; IW: rare forest site. A full list of the samples and their details can be found in Supplementary material S2.xlsx

To further investigate differences in cobalamin production, we measured cobalamin and DMB concentrations in an independent set of 40 soil samples. Representative soil samples were collected from different environments and processed to quantify cobalamin concentrations from both water-leachable and non-water-leachable (i.e., intracellular and/or mineral-bound cobalamin) extracts (Supplementary material S2). Total cobalamins (sum of both water-leachable and non-water-leachable cobalamin) ranged between 0.06 and 6.84 pmol g^−1^ dry soil across all 40 soil samples tested, with an average of 1.19 pmol g^−1^ dry soil (Fig. 5, Supplementary material S2), and with >90% of extractable cobalamins obtained quantitatively and reproducibly with a single extraction (Supplementary material S3; Supplementary information Materials and Methods). Within the total cobalamins pool, the water-leachable fraction generally accounted for a small proportion in all samples (10.1 ± 11.9%; Fig. 5), indicating a strong association between cobalamins and the soil matrix and/or microbial biomass.

**Fig. 6:**
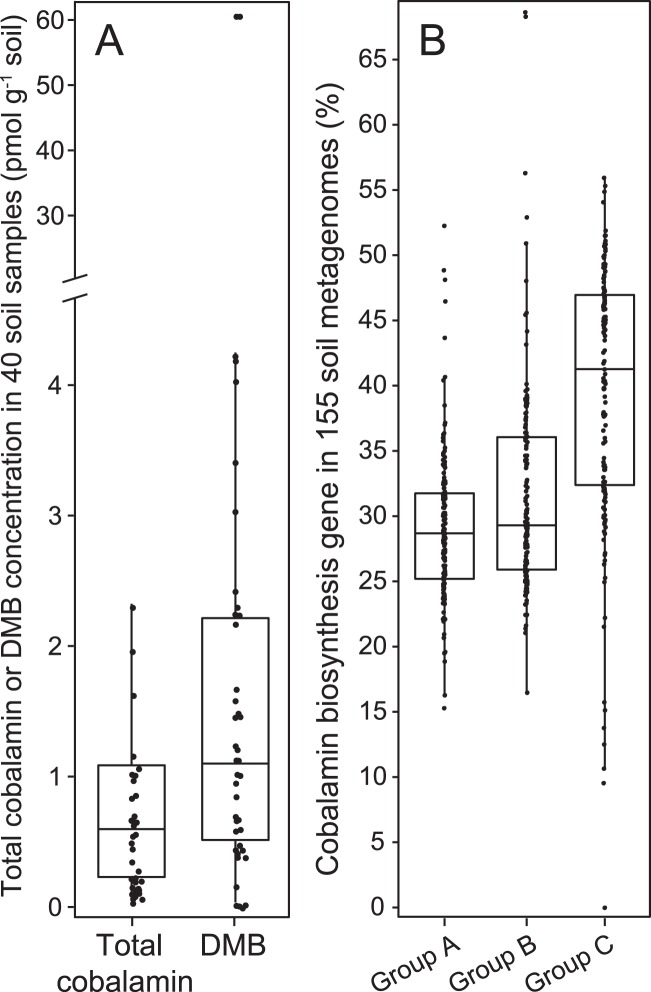
Measured total cobalamin (sum of both water-leachable and non-water-leachable cobalamin) and 5,6-dimethylbenzimidazole (DMB) concentrations in 40 soil samples (**a**), and cobalamin biosynthesis gene summary for 155 soil metagenomes (**b**). Cobalamin-producing genes in **b** were grouped as: Group A, corrin ring biosynthesis; Group B, final synthesis and repair; Group C, 5,6-dimethylbenzimidazole (DMB) synthesis. The 155 soil metagenomes are independent from the 40 soil samples used for cobalamin measurement. The center line in the box plot represents the median value

Similar to total cobalamin, the cobalamin lower ligand (DMB) also dominated in the non-water-leachable pool that contained an average of 82.4 ± 31.8% of the total extractable DMB ligand. The average concentration of DMB was higher than that of total cobalamin (Fig. 6a Supplementary material S2). In one sample, the concentration of DMB was ~40 times that of total cobalamin (***rare*** Charitable Research Reserve IW soil, 15–30 cm). The presence of DMB in soil samples has been reported in a grove soil and creek bank soil [49], and these previous values fall in the range of DMB concentration measured in this study. The presence of DMB concentrations in excess of cobalamin in tested soil samples was consistent with soil metagenomic data (albeit from different soil samples) showing that the DMB biosynthesis gene (group C cobalamin biosynthesis) was more abundant than the other two *cbi*/*cob* gene groups (Fig. 6b; Tukey’s HSD *p* < 0.001). Within the cobalamin pool (OH-, CN-, Ado-, and Me-B_12_), we observed OH-B_12_ as the dominant cobalamin form (relative to CN-, Ado-, and Me-B_12_) based on its concentration in the total pool (sum of both water-leachable and non-water-leachable) (Supplementary material S2), consistent with previous findings from marine systems [19, 23, 50].

We tested for links between microbial diversity, DNA concentration, and cobalamin concentrations in the 40 soil samples analyzed for cobalamin chemistry (Supplementary material S2). When correlating total cobalamin concentration potential with diversity indices for 16S rRNA gene data for all 40 soil samples, significant relationships were observed between cobalamin concentration and Chao1 indices (Spearman’s rank correlation rho = 0.48, *p* < 0.01), richness (observed OTUs; rho = 0.46, *p* < 0.01), and Shannon indices (rho = 0.42, *p* < 0.01). Soil extracted DNA concentrations, a rough estimate of biomass, correlated positively with total cobalamin concentration (Fig. S6; *R*^2^ = 0.57, *p* < 0.01).

## Discussion

Our analysis of terrestrial metagenomes shows that soil-dwelling bacterial taxa with the complete cobalamin-producing pathway represent, on average, less than 10% of the total microbial community based on comparisons to *rpoB* gene relative abundances (Fig. 3). Thus, the data suggest that de novo biosynthesis of cobalamin is a function that is carried out by a relatively small cohort of bacteria and archaea, presumably supplying this essential nutrient to other soil microorganisms, including dominant taxa. Different cohorts of microorganisms with relatively small abundances across diverse soil samples have the genetic potential to carry out this function. This is consistent with cobalamin producers influencing and potentially regulating the growth of other community members by shouldering the high metabolic cost of producing cobalamin [16, 51–53]. Our data indicate that putative cobalamin producers identified in this study serve a “keystone function” in their respective environments. Akin to the notion of keystone species, where individual species exert a disproportionately large effect on the community as a whole, relative to their abundance [54], a keystone function serves a role that is more important than the collective abundances of the genes/species that carry out that function within the community. We argue that in microbial communities, where phylogenetic diversity far exceeds functional diversity, keystone functions may be more relevant as an ecological concept than keystone species.

Based on previous surveys of putative cobalamin-producing phyla [15, 19], *Proteobacteria* were detected as numerically abundant cobalamin producers in both marine [15] and soil environments (Supplementary material S1). The phyla *Cyanobacteria* and *Bacteroidetes*/*Chlorobi* are hypothesized to be abundant pseudocobalamin and/or cobalamin sources in aquatic environments [15, 19], but they did not contribute a significant proportion of genes that encode cobalamin synthesis enzymes. Instead, *Actinobacteria*, *Firmicutes*, and *Acidobacteria* numerically dominated phyla in soil metagenomes that affiliated with gene sequences coding for enzymes that catalyze cobalamin biosynthesis. Despite being abundant cobalamin producers in marine environments (e.g., contributing over 80% of the cobalamin genes in some samples) [15, 19], thaumarchaeotal cobalamin synthesis genes were relatively rare within sampled soil metagenomes (Fig. 2). Nevertheless, because all known thaumarchaeotal cultures produce cobalamin [55], and the per cell cobalamin concentrations for members of the *Thaumarchaeota* in exponential phase can be orders of magnitude higher than for members of the *Proteobacteria* [19], *Thaumarchaeota* may be a more important source of soil cobalamin than gene counts alone would suggest [56]. Similarly, although *Proteobacteria* dominated cobalamin synthesis gene affiliations, this was not necessarily the phylum that contributed most to cobalamin biosynthesis because the genes may not be functional and there is no fixed relationship between gene presence and cellular quotas for cobalamin.

Our results show that, collectively, rare biosphere (<0.1% relative abundance) and intermediate abundance (0.1–5.0% relative abundance) genera are equally likely to be cobalamin producers (~50%) but the most abundant soil genera (>5% relative abundance) generally lack genes coding for cobalamin synthesis (Table 2). Because rare taxa have been demonstrated to mediate nutrient cycles [57] and plant production [58], our results suggest that future experiments should investigate the collective importance of rare cobalamin-producers to microbial community function more broadly. Based on our metagenome analysis, it is still not clear whether, within the same genus, cobalamin biosynthesis can be completed via a collaboration among species within that genus. However, a recent whole genome study of bacteria and *Thaumarchaeota* demonstrated that when a genome contains genes necessary for corrin biosynthesis, it is likely to also have genes for DMB biosynthesis and activation [19]. Although taxonomic resolution is limited at the species level, a similar issue also exists when exploring cobalamin production and/or transport at the genus level. We observed that several abundant genera showed a high proportion of both cobalamin synthesis and transport potentials. For example, *Sphingomonas*, an abundant genus, affiliated with >20% of soil *btuB* and *cob*/*cbi*/*bluB* genes in some soil metagenomes. However, when retrieving representative genomes and examining them at the species level, we noticed either a lack of an annotated *btuB* gene in the genomes or a *btuB* gene with a low annotation score, whereas cobalamin synthesis genes are present in different species, suggesting that individual species probably synthesize or transport cobalamin, but not both. A caveat is that taxonomic assignment for a functional gene requires increased resolution at lower taxonomic ranks, in this case at the species level, to avoid masking by a higher rank. Horizontal gene transfer can also complicate taxonomic classification, which might cause a cobalamin biosynthesis gene to be attributed to the wrong species, and consequently overestimate certain genera.

In addition to measuring cobalamin concentrations in soils, we measured DMB, which has no other known function in cells beyond serving as the lower ligand of cobalamin. We found relatively high concentrations of DMB compared with cobalamin. Group C cobalamin biosynthesis gene abundances were higher than group A and B cobalamin biosynthesis gene abundances (Fig. 6), suggesting greater biosynthesis potential of DMB compared with cobalamin. Because the *bluB* gene codes for the aerobic pathway for DMB synthesis, and is only reported for Gram-negative bacteria and members of the *Thaumarchaeota* [19, 48, 59], it is possible that other microorganisms could employ the anaerobic pathway encoded by *bzaABCDE* genes [59] and not be included in our analysis. Therefore, our estimate of microorganisms with the potential to produce DMB represents a lower limit. Free DMB would offer the potential for cobalamin-like compounds (i.e., pseudocobalamin) present in terrestrial environments to be remodeled into cobalamin. Microorganisms capable of transforming cobalamin-like compounds to cobalamin play a critical role in maximizing the impact of cobalamin production. For example, the purple bacterium, *Rhodobacter sphaeroides*, has been shown to remodel pseudocobalamin with amidohydrolase (CbiZ) and cobinamide-phosphate synthase (CbiB) [20, 60]. It is also possible that free DMB is released during cobalamin degradation, as has been seen in gut bacteria [61], or that DMB is inherently more stable than cobalamin. Nevertheless, our data suggest the possibility that DMB could be readily exchanged among different microorganisms through cross feeding, enabling subsequent remodeling within cells, as has been hypothesized for the gut microbiome [6].

Soil nitrogen cycle microorganisms, which are important for soil primary production, either produce or rely on cobalamin. We found that *Nitrospira* spp., which are diverse and abundant nitrite-oxidizing bacteria [62], were detected in soil metagenomes with the genetic capacity for complete cobalamin synthesis, in agreement with previous reports of genes encoding cobalamin-producing enzymes in the *Candidatus* Nitrospira defluvii genome [63]. Our data demonstrated that *Nitrospira* sequences were associated with ~0.5% of all cobalamin biosynthesis genes across 155 soil metagenomes (Fig. S3), spanning cold desert, desert, forest, grassland, and other terrestrial environments. Genome analysis demonstrated the presence of cobalamin-dependent enzymes involved in porphyrin synthesis and methyl-accepting chemotaxis, suggesting a requirement for this cofactor [2, 63]. The presence of these cobalamin-dependent nitrogen cycling microorganisms that also produce cobalamin suggests links between cobalamin-production and the aerobic nitrogen cycle in diverse soil biomes. This points to a possible mechanism for the loss of cobalamin-producing genes that is mediated by the ubiquity of microorganisms that catalyze N-cycle transformations and simultaneously produce cobalamin for broader community benefit.

Strong positive correlations between extracted DNA and cobalamin concentrations (Fig. S6) imply an importance for cobalamin supply in overall soil microbial community size. Although DNA extract yields from soil samples have been used to represent bacterial biomass [64], we acknowledge that the efficiency of extraction might also be influenced by soil type [65, 66] and DNA from dead cells as so-called relic DNA [67, 68]. However, we note that previous work found no correlation between intracellular cobalamin and microbial biomass in marine samples [19, 50]. We showed that the highest cobalamin concentration in a gram of soil can be two orders of magnitude greater than those measured in a liter of seawater [19], which is likely due to the lower amount of microbial biomass in 1 l of seawater relative to a gram of soil [69]. Although release and uptake mechanisms are not well understood, lower, but quantitatively significant, concentrations of water-leachable relative to non-water-leachable cobalamin in soils suggest that cobalamin is likely efficiently scavenged and relatively stable in soil. It is also possible that a portion of cobalamin in soil is mineral-bound and unavailable to microorganisms.

Cobalamin may be exuded into the soil matrix by living cells or released by cell lysis. In either case, cobalamin producers may play a role that is consistent with the Black Queen hypothesis [70]. Because most bacteria and archaea lack the ability to biosynthesize cobalamin, and thus depend on the small population of cobalamin producers for this cofactor (Fig. 3), cobalamin may play an important role in shaping and supporting soil microbial communities. Microorganisms with genes that encode the cobalamin-dependent methionine synthase (*metH*) alone accounted for ~90% of all microbial community members, almost eightfold more abundant than microbial cobalamin producers. Indeed, Giovannoni proposed that cofactor production by a subset of microbes in a community could be “the most widespread and important example of metabolic outsourcing” [16]. In the marine environment, organic compounds are hypothesized to be exchanged for cobalamin between bacteria and algae [11, 71–73]. The significant positive correlation between cobalamin concentration and diversity indices implies that a greater amount of cobalamin producers but lower amount of cobalamin consumers might foster a more diverse soil microbial community.

As novel cobalamin analogs are being discovered, it is likely that our understanding of how this cofactor relates to soil microbial ecology will continue to expand. For example, nitrocobalamin (NO_2_-cobalamin) was recently discovered in a marine ammonia-oxidizing archaea isolated from an oxygen deficient zone and other low oxygen water samples [56]. Because soil depth influences oxygen availability, future research will target this form of cobalamin in subsurface soil samples and better understand its relevance for AOA within aquatic and terrestrial habitats. In addition, future research should examine cobalamin transport in more detail. A recent study reported that the human gut microbiome contains corrinoid (a group of compounds containing corrin rings; cobalamin being one corrinoid) transporter families that can preferentially capture distinct corrinoids [4]. It would be intriguing to see if soil microorganisms are equipped with similar transport systems. Although relatively abundant in the marine environment [19], psuedocobalamin (i.e., a cobalamin analog with adenine as a lower ligand instead of DMB) was only detected in one soil sample (13CO, CM^2^BL; data not shown). Differences in cobalamin patterns between terrestrial and marine habitats might underpin distinct cobalamin usage mechanisms and consequently metabolisms among microorganisms.

Pioneering studies on cobalamin as a growth factor for soil bacteria in the 1950s observed bacteria that did not grow on nonselective media could recover when supplemented with soil extracts [41–43], and more than half of isolates that could only grow with a soil extract supplement also grew with cobalamin amendment alone [41], implicating cobalamin as a significant microbial growth factor in soil. Our current metagenomic and biochemical perspectives provide strong evidence for an important role played by cobalamin-producing taxa in relation to the much larger overall microbial community in terrestrial environments. Lower ligand remodeling mechanisms are likely to be common among soil microorganisms given the presence of higher concentrations of free DMB than total cobalamin, and a higher relative abundance of DMB synthase encoding genes than those encoding corrin biosynthesis enzymes in soil metagenomes. This study, by quantifying soil cobalamin and identifying the bacteria and archaea that potentially contribute to cobalamin synthesis, transport, dependence, and remodeling in soils, strongly implies that cobalamin producers help maintain an abundant and diverse terrestrial microbial community that, in turn, plays a critical role in the broader health of terrestrial ecosystems.

## Materials and Methods

### Soil metagenomes analysis

We analyzed 155 soil metagenomes available from the Metagenomics RAST Server (MG-RAST; accession numbers listed in Supplementary material S1), ensuring a diverse selection of soil habitats including grassland, agriculture, forest, desert, cold desert, wetland, pasture, herb, and tundra. A set of 12 genes and associated profile HMMs were selected to represent both aerobic and anaerobic cobalamin biosynthesis pathways (Fig. 1; Supplementary material S1), minimizing a potential pathway-specific bias, as described previously for the analysis of marine metagenomes [15, 19]. Marker genes for cobalamin production were classified into three categories (Fig. 1 and Supplementary material S1): group A, corrin ring biosynthesis; group B, final synthesis and repair; group C, DMB synthesis [11, 19]. Taxa included in any of the three groups must show the presence of all marker genes within that group from the same soil metagenome and the taxa with complete cobalamin synthesis pathways were assigned based on the presence of all 12 cobalamin biosynthesis genes in the same taxonomic group from the same soil metagenome. Total microbial communities associated with each metagenome were estimated by quantifying a single-copy gene for the RNA polymerase beta subunit (*rpoB*) [74–76]. The cobalamin transporter gene, *btuB* [45, 77], was selected as a marker for evaluating cobalamin uptake potential. Three cobalamin-dependent enzyme coding genes, *metH*, *mutA*, and *rsmB* [2] were included to further explore cobalamin utilization potential. The input HMM profiles (Supplementary material S1) for *cob*/*cbi*, *bluB*, *btuB*, *metH*, *mutA*, and *rsmB* genes were retrieved from TIGRFAM [78] or Pfam [79], and the phylogenetic marker, *rpoB*, from FunGene [80]. Metagenomic matches to cobalamin genes were identified, quantified, and taxonomically annotated using MetAnnotate [81]. The detailed MetAnnotate setup are summarized in Supplementary information Materials and Methods.

### Soil cobalamin measurement

Soil samples for cobalamin and DMB measurements (Supplementary material S2) were composed of a collection of soils from three different projects: Canadian MetaMicroBiome Library (CM^2^BL) soils covering wide range of soil biomes across Canada [82], two distinctive land-uses along soil profiles (***rare*** Charitable Research Reserve, Cambridge, Ontario) [83], and a pH gradient (4.5–7.5) of agricultural soils (Scottish Agricultural College, Craibstone, Scotland) [84]. Both water-leachable cobalamins and DMB, and non-water-leachable cobalamins and DMB were extracted and measured from all soil samples using a method modified from that developed by Heal et al. [19]. Detailed processes and 16S rRNA gene data sources are summarized in Supplementary information Materials and Methods.

Soil DNA was used for microbial community size estimation in these soils, and was extracted according to the manufacturer protocols with the PowerSoil DNA Isolation Kit (MO BIO, Carlsbad, CA), and quantified with a Qubit 2.0 fluorometer (Invitrogen, Carlsbad, CA).

### Data analysis and visualization

Soil metagenome data were transformed into sample-based relative abundance (dividing gene reads of each taxa over total gene reads in the corresponding soil metagenome) before statistical analysis. All statistical analyses were carried out using R (V 3.2.3). Analysis of variance (ANOVA) was performed to test the effect of environmental factors on gene abundance or cobalamin concentration, followed by post-hoc Tukey HSD test. A Shapiro–Wilk normality test was used to check normality assumption prior to correlation analysis. Spearman’s rank correlations were employed to compare cobalamin concentrations and microbial biomass, and a simple linear regression was used to test for a significant linear relationship. The influence of soil types on cobalamin-producing phyla composition was evaluated by permutational multivariate analysis of variance (adonis) with the *vegan* (V 2.4.3) package in R. In order to test the relationship between cobalamin-producing and consuming taxa in the soil metagenomes studied, Spearman correlation coefficients between the relative abundance of *cob*/*cbi*/*bluB* and *btuB* genes were calculated and compared to the coefficient through 10^5^ permutations to determine significance values. The *phyloseq* (V 1.14.0) [85] R package was used to preprocess OTU tables and calculate diversity indices.

## Supplementary information

Merged supplemental material

Supplemental material S1

Supplemental material S2

Supplemental material S3
