# Supplementary material for: Metagenomic and chemical characterization of soil cobalamin production
Source: ISME J. 2019 Sep 6;14(1):53–66. doi: 10.1038/s41396-019-0502-0 (PMC6908642; doi:10.1038/s41396-019-0502-0)
Supplement: Supplementary file 1 — Merged supplemental material [file 41396_2019_502_MOESM1_ESM.docx]

**Metagenomic and chemical characterization of soil cobalamin production**

**SUPPORTING INFORMATION**

Xinda Lu^1‡^, Katherine R. Heal^2^, Anitra E. Ingalls^2*^, Andrew C. Doxey^1*^, Josh D. Neufeld^1*^

^1^Department of Biology, University of Waterloo, ON, Canada

^2^School of Oceanography, University of Washington, WA, USA

^‡^Current address: Department of Civil and Environmental Engineering, Massachusetts Institute of Technology, Cambridge, MA, USA

^*^Corresponding authors: Anitra E. Ingalls, Andrew C. Doxey, and Josh D. Neufeld

E-mail: aingalls@uw.edu; acdoxey@uwaterloo.ca; jneufeld@uwaterloo.ca

Keywords: cobalamin, soil, computational metagenome, microbial ecology

**Competing interests**

The authors declare no competing interests.

**SI Materials and Methods**

*Taxonomic classification using functional genes*

MetAnnotate [1], was used to mine for selected marker genes (Supplemental material S1) in unassembled soil metagenomes, before which FragGeneScan was used with default setting to predict genes in short reads [2]. Each HMM is searched via HMMsearch [3] against NCBI RefSeq [4] release 80 for reference homologs, and against the 155 soil metagenomes for homologs, with an *E*-value threshold of 10^-6^. Taxonomic classifications were made based on the best hits in the RefSeq database, using USEARCH [5] with a 50% minimum amino acid identity threshold. Metagenome HMM hit counts were normalized by HMM length. An OTU table, showing the number of gene reads, was generated for each corresponding gene.

*Soil samples for cobalamin measurement*

The ***rare*** Charitable Research Reserve soil samples were retrieved from three different depths (L1: 0-15 cm, L2: 15-30 cm, L3: 30-45 cm) for each land use type. The microbial community compositions of these soils have been reported elsewhere [6–9], but all representative 16S rRNA gene reads from these projects were re-classified using RDP Naive Bayesian rRNA Classifier (version 2.11), with default settings [10].

*Soil cobalamin and DMB extraction and UPLC/MS measurement*

Field moist soil (0.5 g) was added to 15-ml tubes (VWR, Radnor, PA), with approximately 2 mL of a mixture of 100 µm, 400 µm, 1.4 mm, and 4.0 mm glass beads (OPS Diagnostics). For water-leachable extraction of cobalamins and DMB, each tube was filled with 7 ml of 2 M KCl solution and mixed gently every 10 minutes at room temperature for 60 minutes. The tubes were centrifuged at 4000 rpm at 4°C for 15 minutes. Supernatants were transferred into 24-ml glass vials and stored at -20°C until desalting and downstream cobalamin analysis. Following the removal of the water-leachable portion, remaining DMB and cobalamins were extracted using three solutions with decreasing polarity. Solutions used were prepared fresh and the recipe of each solution can be found at the end of this section. Samples were initially subjected to beadbeating three times in 7 ml of solution A for 90 seconds; samples were kept at -20°C for 30 minutes in between beadbeating rounds. Samples were centrifuged at 4000 rpm, 4°C for 15, and the supernatant was transferred to 60-ml glass vials. Next, 7 ml solution B was added into pelleted soil after removing supernatant, bead beat for 90 seconds, stored at -20°C for 5 minutes, and centrifuged at 4000 rpm for 15 min at 4°C. Supernatants were added to the same 60-ml glass vials. Extraction was repeated with another 7 ml solution B and supernatant transferred to the same vial, adding up the final volume to 21 ml for each glass vial. Another three rounds of bead beating and centrifugation were carried out using solution C (7 ml each time, 21 ml in total), and the 21 ml extracts transferred to 60-ml vials, followed by three, 7 ml MeOH extractions. Each MeOH extract was combined with the 21 ml extracts in the 60-ml vials. Samples in 60-ml vials were dried down overnight under clean N_2_ at no more than 35°C. Finally, samples were reconstituted with 3 ml Milli-Q H_2_O, sonicated and vortexed. ﻿

Desalting was carried out following a protocol similar to that used to extract cobalamin from marine water samples [11]. For each sample (water-leachable or non-water-leachable) a C18-500 mg SPE column (Waters Sep-Pak) was conditioned by passing through 5 ml methanol, followed by a rinse with 5 ml Milli-Q H_2_O, both via gravity. Samples were loaded to SPE columns via gravity, followed by two rinses with 3 ml of Milli-Q H_2_O and 3 ml of solution D, respectively. After the rinses, samples were eluted with 5 mL of solution E and collected into 12-ml borosilicate centrifuge tubes. Tubes were dried down under vacuum at low heat (no more than 35°C). Samples were reconstituted using 380 µl solution F and 20 µl vitamin injection standard mix, sonicated for 2 minutes, and then syringe filtered into labeled amber I-class vials. All extraction steps were carried out with minimum light and covered in aluminum foil when possible to avoid light exposure. We analyzed the samples using ultra performance liquid chromatography – mass spectrometry (UPLS/MS) using the exact conditions and vitamin internal standards as previously published [11] for the cobalamins. For DMB, we used MS conditions reported elsewhere [12], paired with the UPLC conditions for the cobalamin analysis. Data were extracted, integrated, and processed through in-house quality control and normalization as reported by Boysen and colleagues [12]. This methodology was developed in natural marine samples and accounts for variable matrix effect by using internal standards and employs standard addition for quantification [11–13], making it appropriate for the complex organic matrix in these samples. Total cobalamins and DMB is the summed value of all detected forms of cobalamin or DMB in both the water-leachable and non-water-leachable fractions of the soil.

Solutions A-E were as follows, using Omnisolve grade solutions. Solution A: 50% methanol, 50% 0.2 M KCl (vol/vol); solution B: 50% methanol, 50% Milli-Q H_2_O (vol/vol); solution C: 40% acetonitrile, 40% methanol, 20% Milli-Q H_2_O, 0.1% formic acid (vol/vol); solution D: 10% methanol, 90% Milli-Q H_2_O (vol/vol); solution E: 80% methanol, 20% Milli-Q H_2_O (vol/vol); solution F: 20 mM ammonium formate and 0.1% formic acid in H_2_O.

We tested the reproducibility of this extraction in two different soil types (***rare*** forest and agriculture). We evaluated the reproducibility of our measurements in both the water-leachable and non-water-leachable fraction of cobalamin and DMB (Supplemental material S3); considering the heterogeneous nature of soil, we considered the ±32% (for water-leachable) and ±23% (for non-water-leachable) reproducibility acceptable. To assess our extraction procedure for extraction efficiency, we performed a double extraction, that is, an extraction of the exact type on soils after a full extraction procedure. In our second extraction of the soils, we never saw more than 7% of the B_12_ or DMB as the first extraction (Supplemental material S3), suggesting that the vast majority of the extractable cobalamins and DMB were removed with our standard extraction. Without any consensus standards, it is difficult to know if the extraction procedure used here the most effective, but the reproducibility of our tests suggests it is a consistent methodology that yields comparable data between samples.

# **References**

1. Petrenko P, Lobb B, Kurtz DA, Neufeld JD, Doxey AC. MetAnnotate: function-specific taxonomic profiling and comparison of metagenomes. *BMC Biol* 2015; **13**: 1–8.

2. Rho M, Tang H, Ye Y. FragGeneScan: predicting genes in short and error-prone reads. *Nucleic Acids Res* 2010; **38**.

3. Eddy SR. Profile hidden Markov models. *Bioinformatics* 1998; **14**: 755–763.

4. O’Leary NA, Wright MW, Brister JR, Ciufo S, Haddad D, McVeigh R, et al. Reference sequence (RefSeq) database at NCBI: current status, taxonomic expansion, and functional annotation. *Nucleic Acids Res* 2016; **44**: D733–D745.

5. Edgar RC. Search and clustering orders of magnitude faster than BLAST. *Bioinformatics* 2010; **26**: 2460–2461.

6. Neufeld J, Engel K, Cheng J, Moreno-Hagelsieb G, Rose D, Charles T. Open resource metagenomics: a model for sharing metagenomic libraries. *Stand Genomic Sci* 2011; **5**: 203–210.

7. Lu X, Seuradge BJ, Neufeld JD. Biogeography of soil *Thaumarchaeota* in relation to soil depth and land usage. *FEMS Microbiol Ecol* 2017; **93**: fiw246–fiw246.

8. Bartram AK, Jiang X, Lynch MDJ, Masella AP, Nicol GW, Dushoff J, et al. Exploring links between pH and bacterial community composition in soils from the Craibstone Experimental Farm. *FEMS Microbiol Ecol* 2014; **87**: 403–415.

9. Seuradge BJ, Oelbermann M, Neufeld JD. Depth-dependent influence of different land-use systems on bacterial biogeography. *FEMS Microbiol Ecol* 2017; **93**: fiw239–fiw239.

10. Wang Q, Garrity GM, Tiedje JM, Cole JR. Naïve Bayesian classifier for rapid assignment of rRNA sequences into the new bacterial taxonomy. *Appl Environ Microbiol* 2007; **73**: 5261–5267.

11. Heal KR, Carlson LT, Devol AH, Armbrust EV, Moffett JW, Stahl DA, et al. Determination of four forms of vitamin B_12_ and other B vitamins in seawater by liquid chromatography/tandem mass spectrometry. *Rapid Commun Mass Spectrom* 2014; **28**: 2398–2404.

12. Boysen AK, Heal KR, Carlson LT, Ingalls AE. Best-matched internal standard normalization in liquid chromatography–mass spectrometry metabolomics applied to environmental samples. *Anal Chem* 2018; **90**: 1363–1369.

13. Heal KR, Qin W, Ribalet F, Bertagnolli AD, Coyote-Maestas W, Hmelo LR, et al. Two distinct pools of B_12_ analogs reveal community interdependencies in the ocean. *Proc Natl Acad Sci USA* 2017; **114**: 364–369.

**
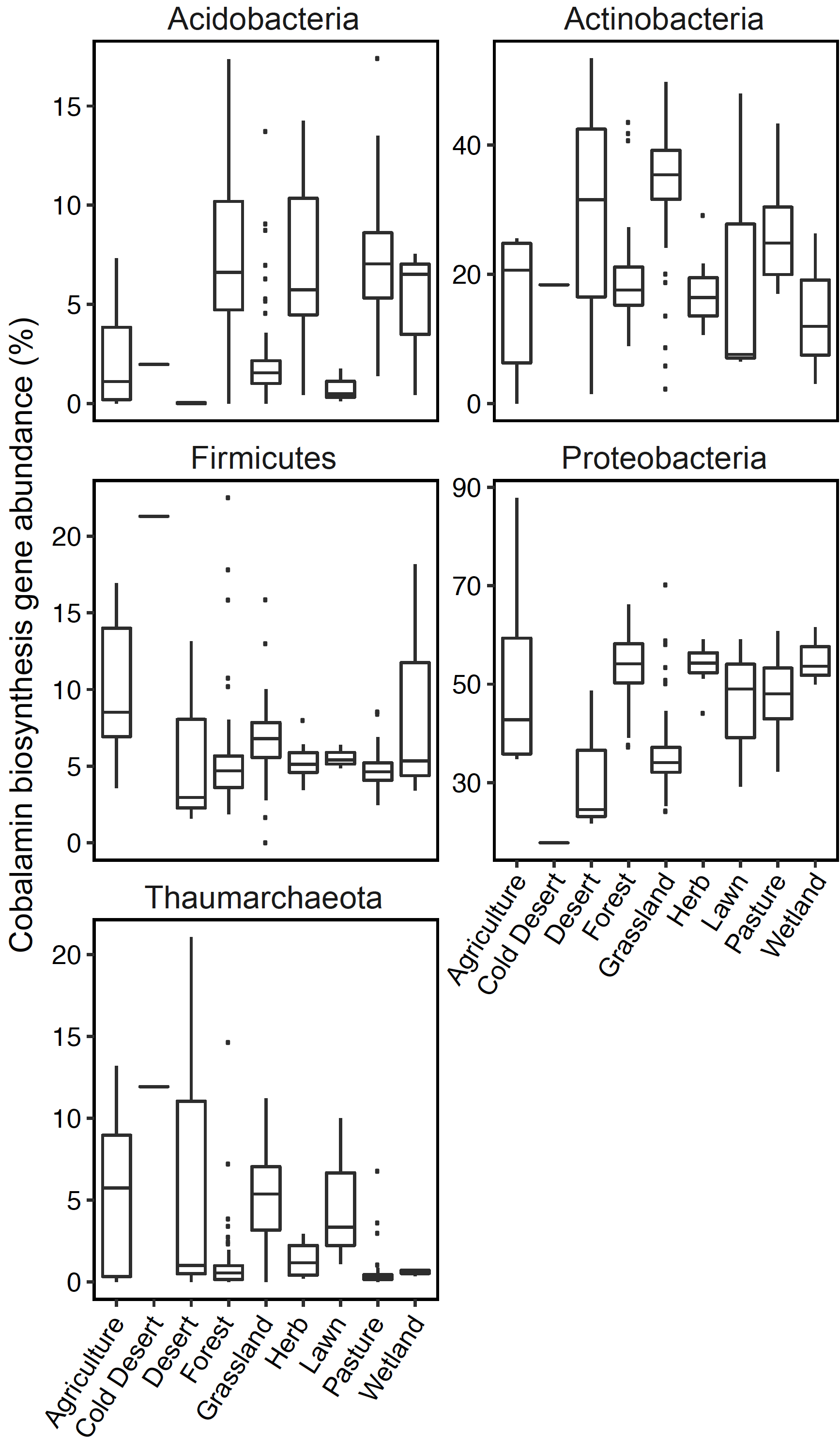
Supplementary Figures**

Figure S1. Relative contributions of each of the major taxa to cobalamin biosynthesis enzyme coding genes in different soil types. Note that only one cold desert sample was included.


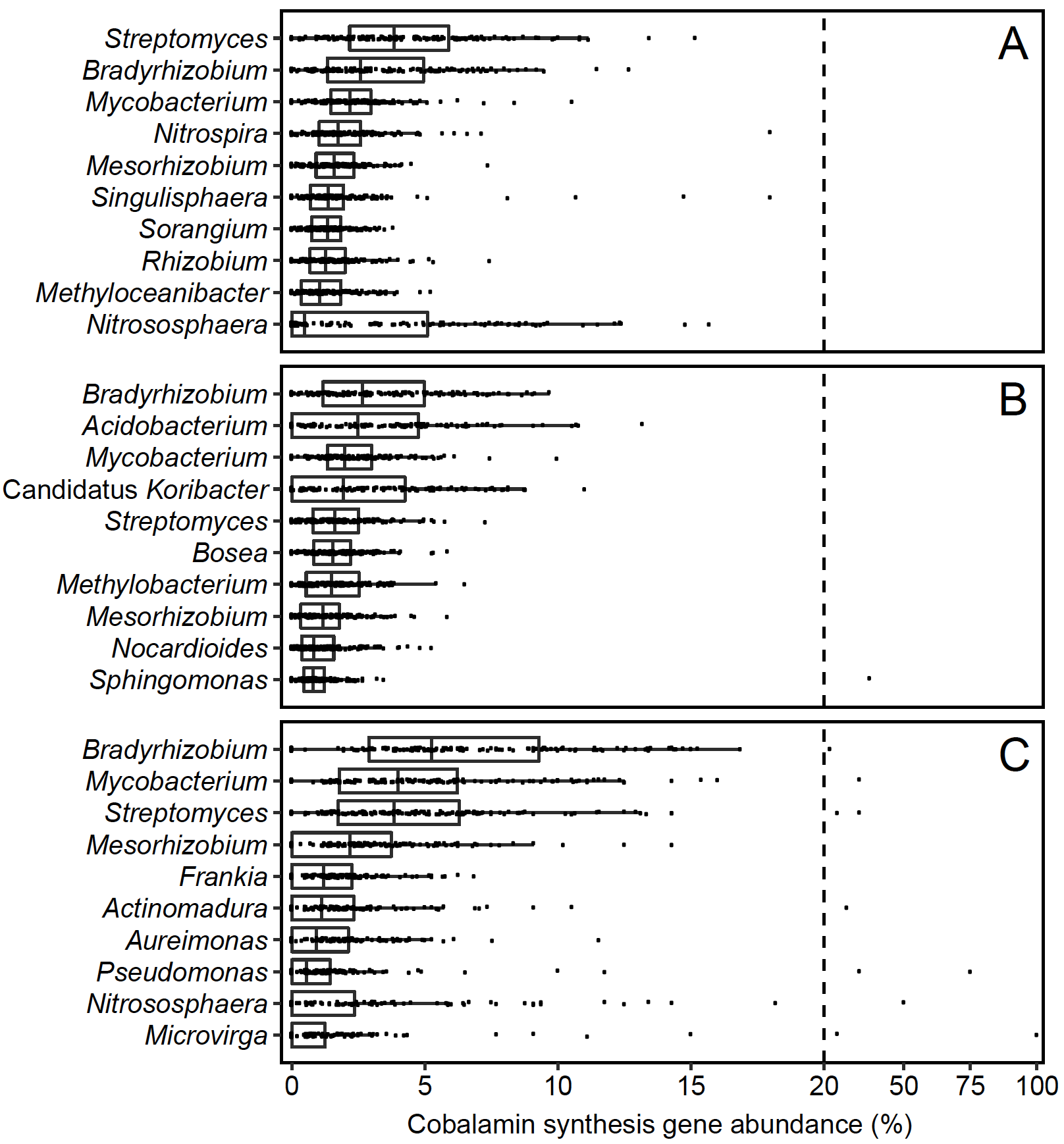


Figure S2. Top genera contributing to cobalamin biosynthesis genes HMM hits. From (A) to (C): Group A, corrin ring biosynthesis; Group B, final synthesis and repair; Group C, 5,6-dimethylbenzimidazole (DMB) synthesis.


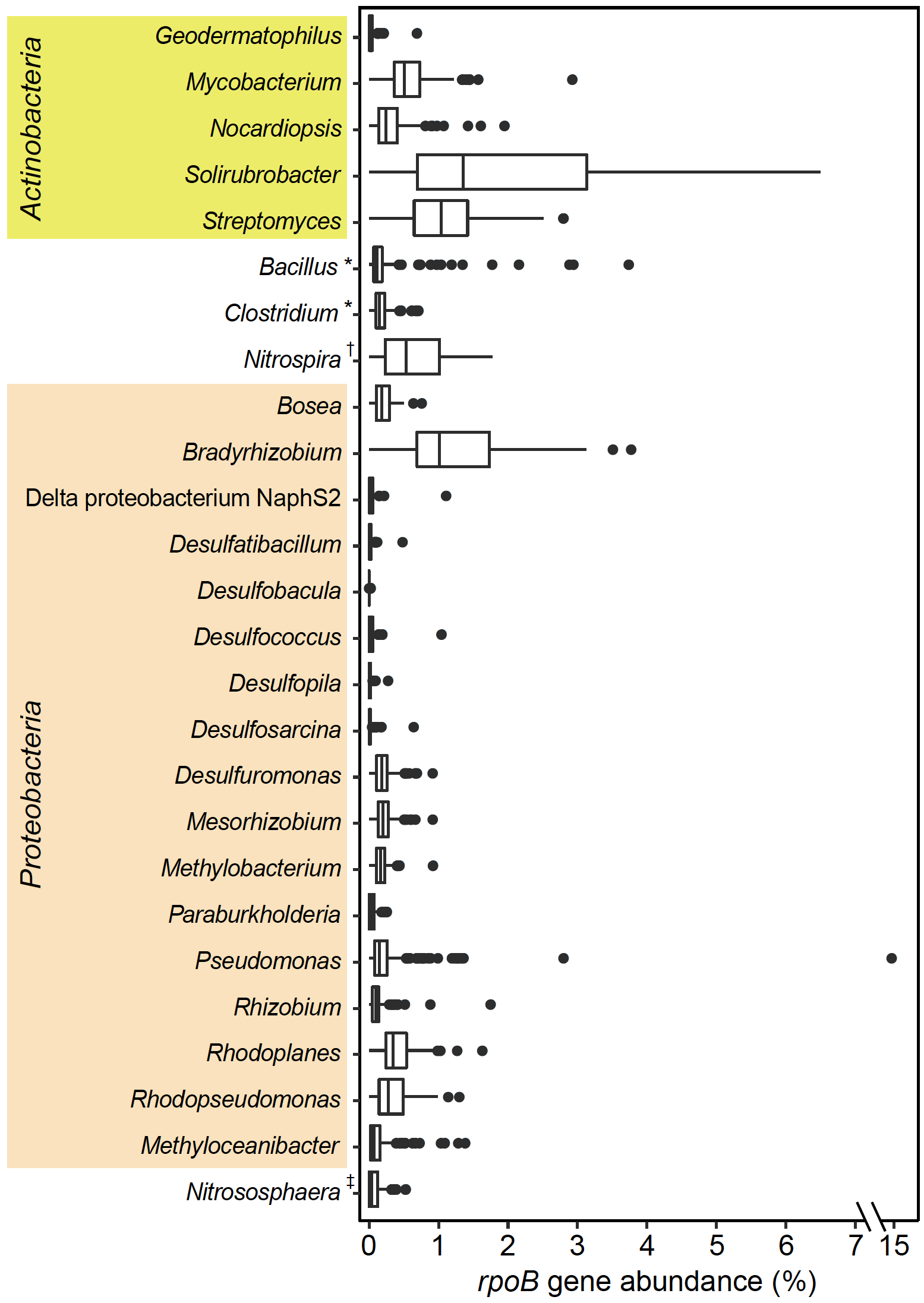


Figure S3. Relative abundance of genera with complete cobalamin synthesis pathways in each of the 155 soil metagenomes based on *rpoB* HMM hits. The corresponding taxa are shown on the left side. Due to limited space, phylum names are not shown for genera: ^*^*Bacillus* and ^*^*Clostridium* (phylum *Firmicutes*); ^†^*Nitrospira* (phylum *Nitrospirae*); ^‡^*Nitrososphaera* (phylum *Thaumarchaeota*).


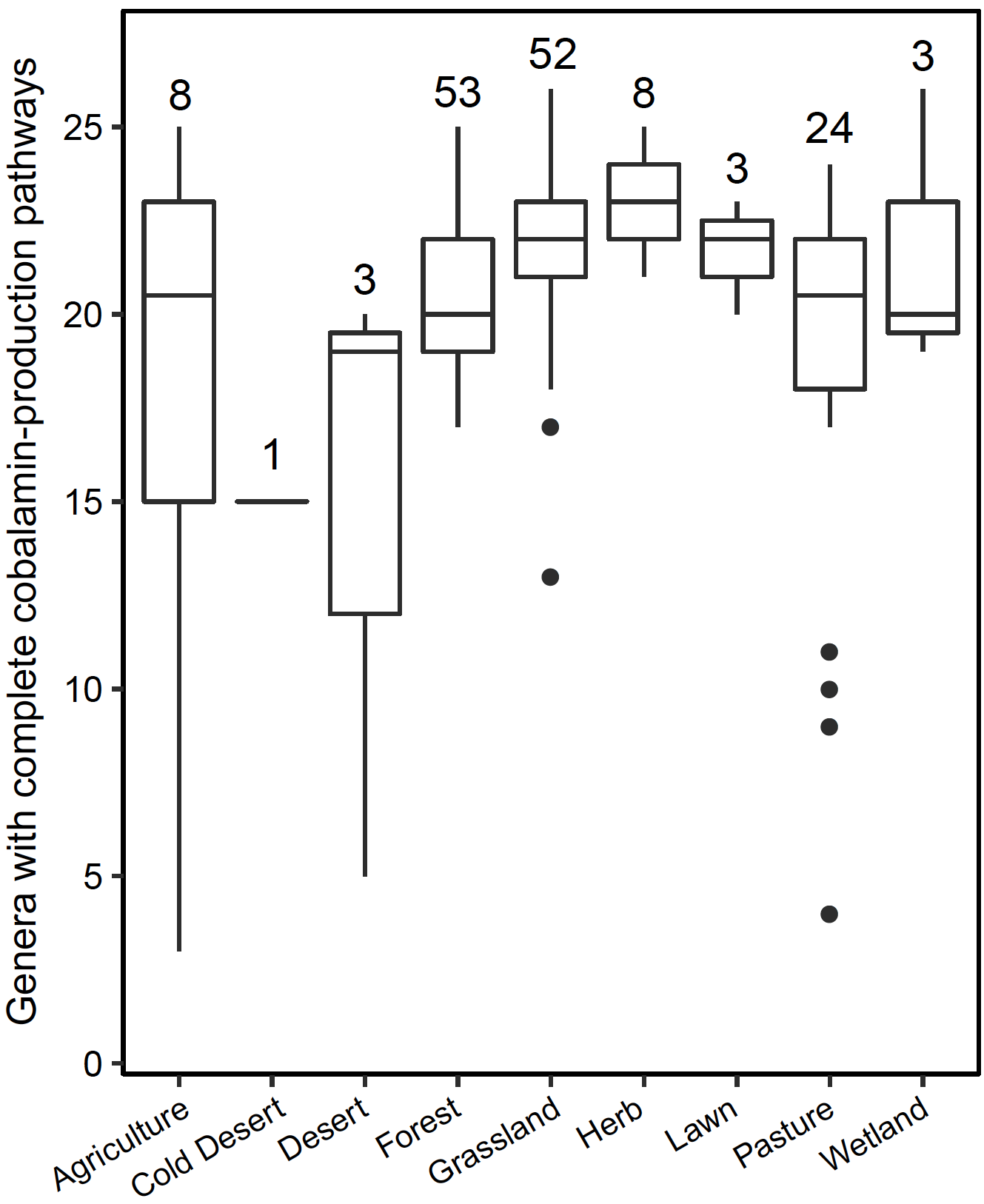


Figure S4. Richness (number of genera) for potential complete cobalamin-producing genera in each of the 155 soil metagenomes, summarized by land use type. The number of soil metagenomes (*n*) included in each land use is shown above each corresponding box. Note that only one cold desert sample was included.


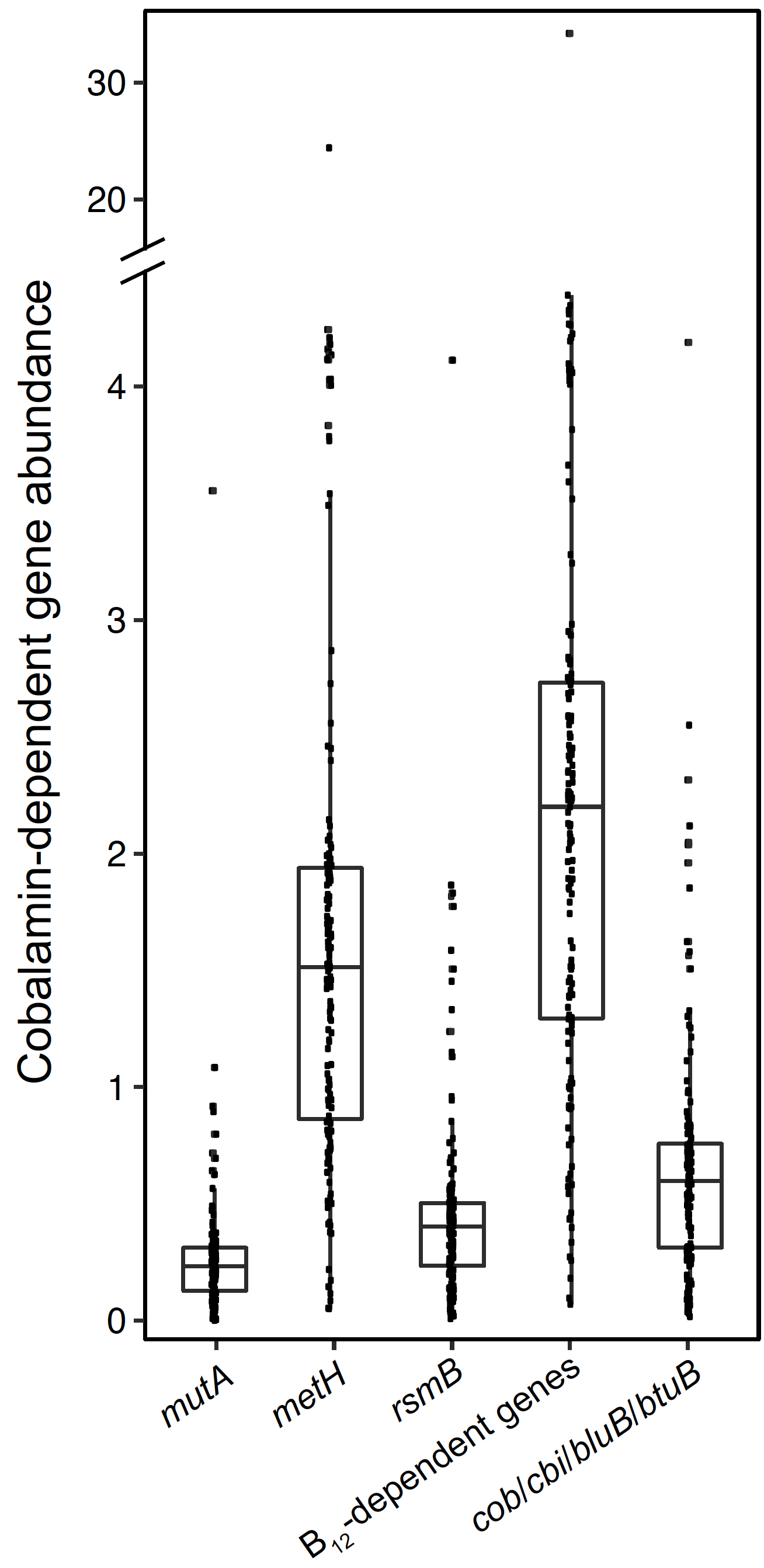


Figure S5. The normalized read abundance of each individual gene for cobalamin-dependent processes (i.e., *mutA*, *metH*, and *rsmB*), sum of these three genes for cobalamin-dependent processes (B_12_-dependent genes), and abundance of those genes encoding cobalamin synthesis and transport (*cob*/*cbi*/*bluB* and *btuB* genes). The read abundances for each gene are normalized to HMM length.


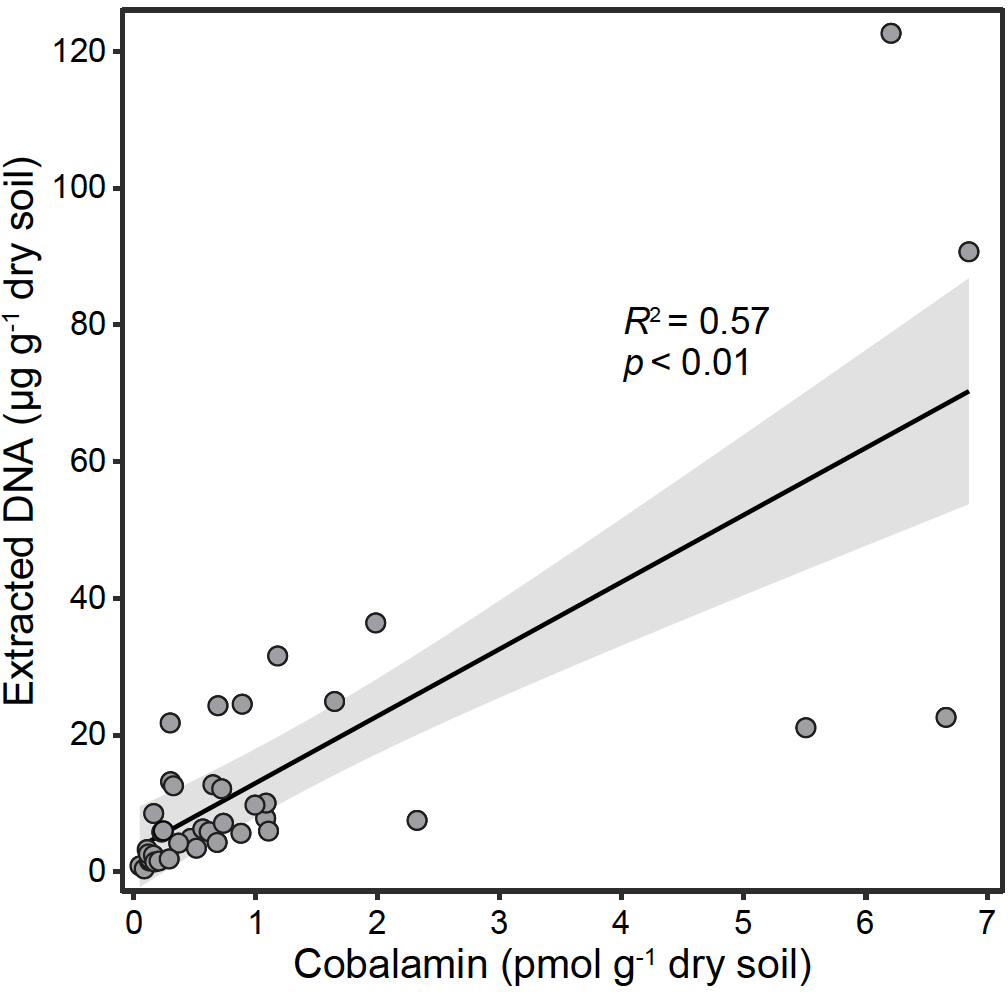


Figure S6. Scatter plot showing positive relationship (simple linear regression, *R*^2^ = 0.57) between total cobalamin concentration and microbial biomass (i.e., DNA yield) across 40 soil samples collected for CM^2^BL, ***rare*** Charitable Research Reserve, and Craibstone pH plots.
